# Kampo Medicine Promotes Early Recovery From Coronavirus Disease 2019-Related Olfactory Dysfunction: A Retrospective Observational Study

**DOI:** 10.3389/fphar.2022.844072

**Published:** 2022-03-30

**Authors:** Rie Ono, Ryutaro Arita, Shin Takayama, Akiko Kikuchi, Minoru Ohsawa, Natsumi Saito, Satoko Suzuki, Tadashi Ishii

**Affiliations:** ^1^ Department of Kampo Medicine, Tohoku University Hospital, Sendai, Japan; ^2^ Department of Education and Support for Regional Medicine, Tohoku University Hospital, Sendai, Japan; ^3^ Department of Anesthesiology and Perioperative Medicine, Tohoku University Hospital, Sendai, Japan; ^4^ Department of Kampo and Integrative Medicine, Tohoku University Graduate School of Medicine, Sendai, Japan; ^5^ Department of Obstetrics and Gynecology, Tohoku University Hospital, Sendai, Japan

**Keywords:** early recovery, kampo, olfactory dysfunction, promotes, coronavirus disease 2019, traditional medicine

## Abstract

**Background:** Olfactory dysfunction is a common symptom in patients with coronavirus disease 2019, and it significantly deteriorates patients’ quality of life. Effective treatments remain unknown.

**Purpose:** To assess the effect of Japanese traditional (Kampo) medicine on coronavirus disease 2019-related olfactory dysfunction.

**Study Design:** Retrospective observational study.

**Methods:** In total, 87 patients aged ≥18 years with coronavirus disease 2019 and severe dysosmia or anosmia (Numeric Rating Scale, ≥7) at isolation facilities in Miyagi Prefecture, Japan, were enrolled from October 2020 to March 2021. Patients were divided into the Kampo group (*N* = 52) and the control group (*N* = 35) based on the treatment received. Changes in Numeric Rating Scale scores were evaluated at the first visit and 2 weeks after.

**Results:** The median reduction in the olfactory dysfunction score at both 1 and 2 weeks after the first visit was significantly greater in the Kampo group (6 and 8, respectively; *p* = 0.03) than in the control group (3 and 7, respectively; *p =* 0.04). We defined improvement in olfactory dysfunction as a median reduction in the olfactory dysfunction score of ≥5. Multiple logistic regression analysis demonstrated that only Kampo treatment was significantly associated with improvement in olfactory dysfunction.

**Conclusion:** This study suggests that Kampo medication promotes early recovery from coronavirus disease 2019-related olfactory dysfunction.

## Introduction

Coronavirus disease 2019 (COVID-19), caused by severe acute respiratory syndrome coronavirus 2 (SARS-CoV-2), which was discovered in Wuhan (Hubei, China) in late 2019, remains a threat to human life (World Health Organization, 2019). As of November 2021, more than 248 million cases of COVID-19 had been identified worldwide, with more than 5 million deaths recorded (COVID-19 dashboard by the Center for Systems Science and Engineering at John Hopkins University, 2021). The Centers for disease Control and Prevention have reported the following clinical manifestations of COVID-19 that appear 2–14 days after exposure to the virus: fever, chills, cough, shortness of breath or difficulty breathing, fatigue, muscle or body aches, headache, new loss of taste or smell, sore throat, congestion or runny nose, nausea or vomiting, and diarrhea (Symptoms of COVID-19, 2021).

Olfactory dysfunction (OD) has been reported as a characteristic symptom of COVID-19. Many viruses can lead to OD through an inflammatory reaction of the nasal mucosa and the development of rhinorrhea (Deems et al., 1991). However, COVID-19-related OD is characterized by sudden onset with or without other symptoms, and it can appear before other symptoms (Kaye et al., 2020); hence, an impaired sense of smell and/or taste is considered a diagnostic criterion for COVID-19. Moreover, the prevalence of OD in patients with COVID-19 was reported to be nearly three times as high as OD in those with other viral infections (Menni et al., 2020; Yan et al., 2020). Previous studies reported the recovery from OD in many patients within a month following the onset of OD (D’Ascanio et al., 2021; Lechien et al., 2020). However, some studies have reported long-term persistence of OD (Bertlich et al., 2021; Bussière et al., 2021; Seo et al., 2021). OD results in reduced quality of life (Temmel et al., 2002); however, effective treatments are limited (Addison et al., 2021). Therefore, early recovery from OD is of great benefit to patients. Previously, we demonstrated in a case series that Kampo medicine, kakkontokasenkyushin’i (KKTSS), could be a therapeutic option for treating COVID-19-related OD (Takayama et al., 2021a). We demonstrated the potential effects of Kampo medicines against SARS-CoV-2 (Arita et al., 2020). Integrated medicine using traditional medicine was shown to contribute to potential treatments for COVID-19 and its related symptoms (Liu et al., 2020); however, there are no studies on the effects on olfactory function. We used Japanese traditional medicine, Kampo medicine, according to the symptoms and conditions of patients with COVID-19. We believe that Kampo medicine might have a potential beneficial effect on the recovery of olfactory function.

Therefore, this retrospective observational study aimed to investigate the treatment effect of Kampo medicine on the improvement in COVID-19-related OD.

## Materials and Methods

### Ethics

The study was conducted in accordance with the principles of the Declaration of Helsinki and Tokyo for humans and was approved by the ethical committee of Tohoku University (Miyagi, Japan) (approval number: 2021-1-447).

### Study Design

In Japan, patients with laboratory-confirmed COVID-19 who were asymptomatic or had mild symptoms were observed at isolation facilities. Nurses assessed the vital signs of the patients (oxygen saturation, pulse rate, and body temperature), and their symptoms were assessed using Numeric Rating Scale (NRS) scores twice daily based on a questionnaire [the total score ranged from 0 (no symptoms) to 10 (intolerable symptoms)]. Medical doctors visited the isolation facilities to treat patients with COVID-19 in Miyagi Prefecture. Patients with persistent symptoms were examined and prescribed medication, such as antipyretics, antitussive drugs, and/or Kampo medicines, based on their condition and symptoms at the discretion of the examining doctor under the Japanese Health Insurance System. Kampo medicines were selected according to patients’ clinical course and symptoms. For example, kakkonto (KKT) was used for acute fever and chills with slight nasal symptoms; KKTSS was used for acute fever and chills with remarkable nasal symptoms; shosaikotokakikyosekko (SSKKS) was used for subacute fever with pharyngeal symptoms; KKT combined with SSKKS was used for acute to subacute fever with bronchitis. The prescription decisions were made by the physicians depending on the patients’ clinical course and symptoms. Kampo medicines are approved for ethical use by the Ministry of Health, Labour, and Welfare, and their manufacturing methods, as well as components and quantity, comply with the Japanese Pharmacopoeia. The term “ethical use” in this context implies that medicine can only be dispensed to patients with a prescription from a doctor, and the expenses are covered by the National Health Insurance. The Japanese Pharmacopoeia (The Japanese Pharmacopoeia, seventeenth edition, English version, 2021) officially defines the origin and description of the listed crude drugs and Kampo extracts and elaborates on their limited values and testing methods. Detailed information on each medication is available on STORK (http://mpdb.nibiohn.go.jp/stork/). Similarly, detailed information on the production and quality control systems for Kampo products is available on the Tsumura website (https://www.tsumura.co.jp/english/kampo/07.html).

This retrospective observational study was conducted at isolation facilities in Miyagi Prefecture in Japan between 1 October 2020, and 31 March 2021. The inclusion criteria were as follows: age ≥18 years, underwent a medical examination, and had severe dysosmia or anosmia defined by an NRS score of 7–10 on the first visit. The exclusion criteria were severe pneumonia or the inability to determine the NRS score for OD 1 and 2 weeks after the first visit.

Eligible participants were divided into two groups: the Kampo group that included patients who were administered Kampo or Kampo combined with Western medication and the control group that included patients who were administered only Western medication or no medication.

### Clinical Outcomes

The endpoints were the changes in NRS scores for OD 1 and 2 weeks after the first visit. We also collected patients’ clinical data and characteristics from observational records of the isolation facilities and medical records from Tohoku University Hospital.

### Statistical Analysis

We compared patient background data using the chi-square test for binary variables and the Mann-Whitney U test for continuous variables. We subsequently conducted the Wilcoxon rank-sum test to compare changes in NRS scores for OD between the two groups. Univariate logistic regression analysis was performed to calculate adjusted odds ratios (ORs) with 95% confidence intervals (CIs) of patient background and type of medication for improvement in OD, which was defined as reduction in NRS score (∆OD) of more than the median value in 1 week after the first visit. Finally, multiple logistic regression analysis was conducted to investigate factors associated with an improvement in OD.

Data are presented as median, range, and percentage, as appropriate. *p*-values of <0.05 were considered statistically significant. All statistical analyses were conducted using R Statistical Software (version 4.0.5) (The R Foundation for Statistical Computing, Vienna, Austria).

## Results

**FIGURE 1:**
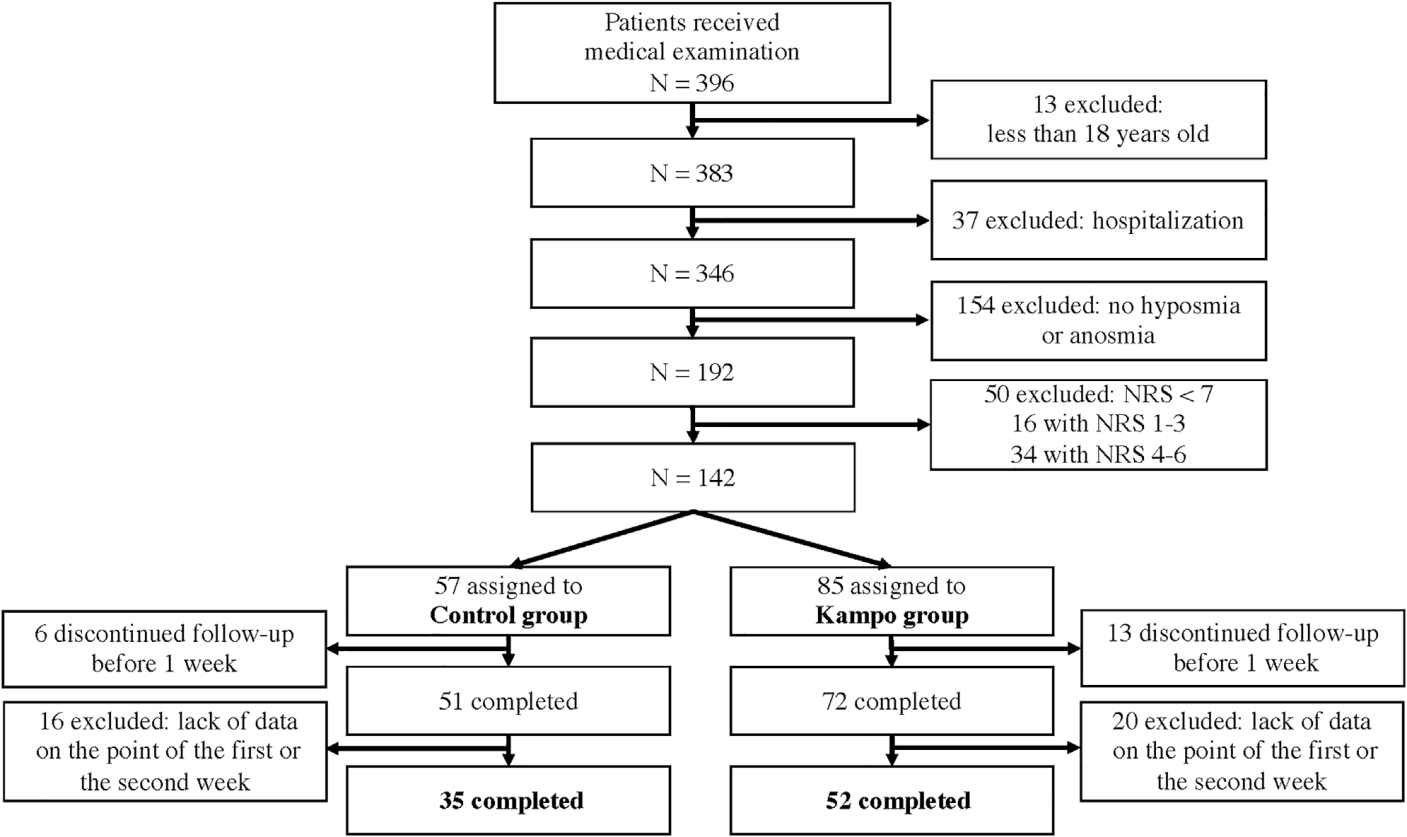
Participant flow diagram.

Overall, 87 eligible patients were included (Figure 1); 52 patients were enrolled in the Kampo group and 35 in the control group. The clinical characteristics are shown in Table 1. The proportion of patients with a current or past history of asthma was significantly higher in the control group than in the Kampo group (*p* = 0.04). The median age of the control group (34 years) tended to be younger than that of the Kampo group (39.5 years) (*p* = 0.052).

**TABLE 1:** Characteristics of study participants.

	Control group (*N* = 35)		Kampo group (*N* = 52)		*p value*
Age [years old, median (range)]	34	(20–69)	39.5	(22–67)	0.05
Sex [N (%)]					
Male	17	−48.6	21	−40.4	0.45
Female	18	−51.4	31	−59.6	
Smoker [N (%)]	12 (NA = 2)	−34.3	12 (NA = 1)	−23.1	0.2
Comorbidities. [N (%)]					
Rhinitis	10	−28.6	19	−36.5	0.87
Asthma	7	−20	3	−5.8	0.04
Diabetes mellitus	1	−2.9	2	−3.8	0.8
Neurological disorder	0	0	1	−1.9	
Psycological disorder	5	−14.3	3	−5.8	0.18
Hypertention	0	0	4	−7.7	0.09
Dyslipidemia	1	−2.9	4	−7.7	0.22
Symptoms. [N (%)]					
Dysgeusia	19	−54.3	32	−61.5	0.5
Nasal obstructions or nasal discharge	20	−57.1	22	−42.3	0.17
Duration from onset of COVID-19 to medical examination [days, median (range)]	7	(4–18)	7	(4–17)	0.83
Duration from onset of OD to medical examination [days, median (range)]	3	(0–13)	3	(0–10)	0.53

NA; noblt applicable.

**FIGURE 2:**
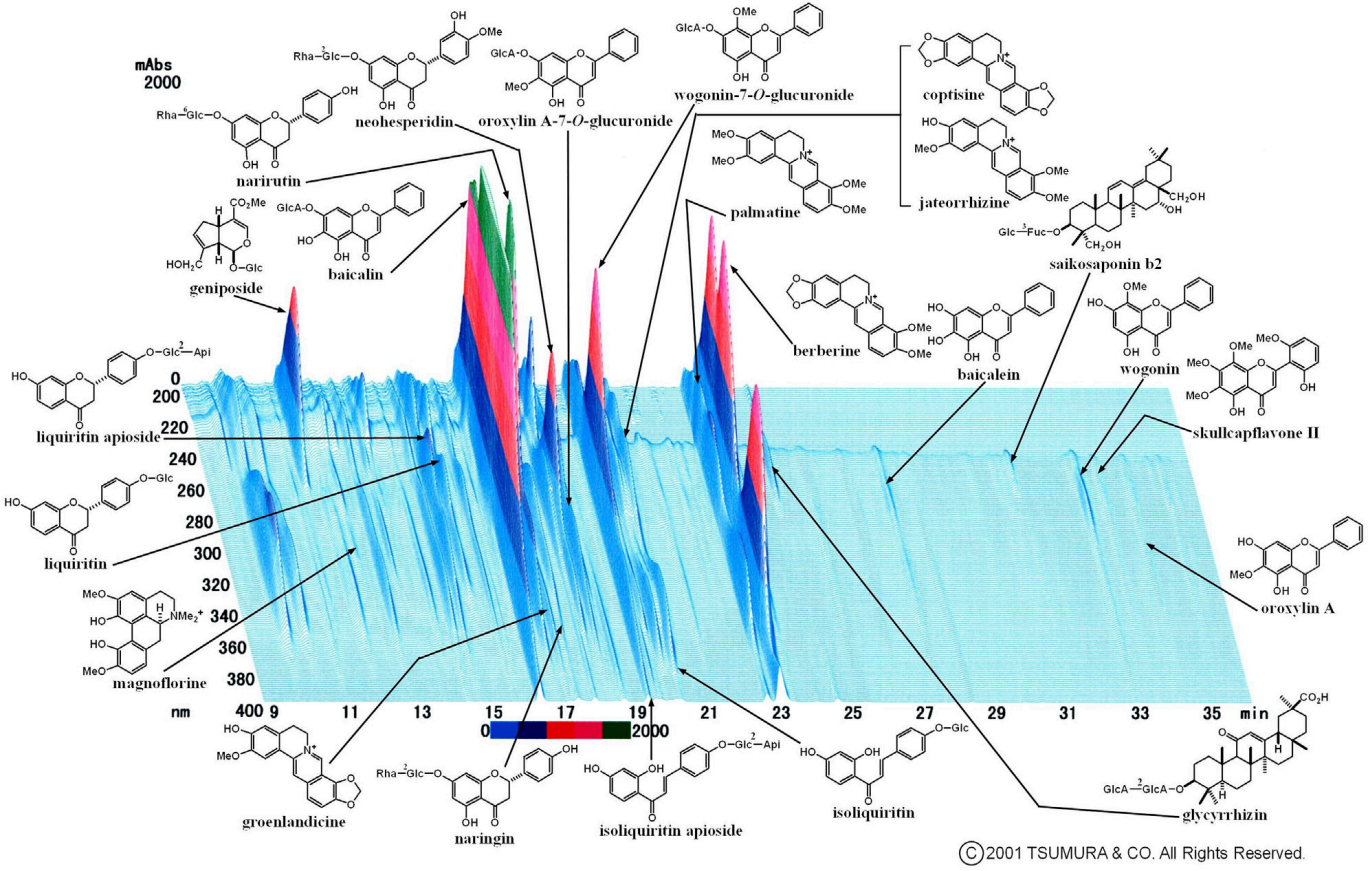
Three-dimensional high-performance liquid chromatography fingerprints of Keigairengyoto Extract Granules for Ethical Use (Tsumura & Co.).

The prescribed medications are described in Table 2. In the control group, 24 patients (68.6%) received Western medication and 11 (31.4%) received no medication. In the Kampo group, 24 patients (46.2%) were treated with Kampo medication alone and 28 (53.8%) were treated with a combination of Kampo and Western medications. The frequently used Western medications were acetaminophen (20 patients; 23.0% in both groups) and dimemorfan phosphate (15 patients; 17.2% in both groups). The frequently used Kampo formulas were keigairengyoto (KRT) (16 patients; 30.8%), SSKKS (14 patients; 26.9%), KKTSS (11 patients; 21.2%), and KKT (8 patients; 15.4%). KRT, which was the most frequently used Kampo medicine in this study, is composed of 17 crude drugs. The current prescription of KRT was KRT Extract Granules for Ethical Use (Tsumura & Co.) (Figure 2), wherein 7.5 g of KRT extract granules contain a dried extract of 17 crude drugs: Japanese Pharmacopoeia [JP] Scutellaria Root (1.5 g), JP Phellodendron Bark (1.5 g), JP Coptis Rhizome (1.5 g), JP Gardenia Fruit (1.5 g), JP Rehmannia Root (1.5 g), JP Peony Root (1.5 g), JP Cnidium Rhizome (1.5 g), JP Japanese Angelica Root (1.5 g), JP Platycodon Root (1.5 g), JP Glycyrrhiza (1.0 g), JP Schizonepeta Spike (1.5 g), JP Forsythia Fruit (1.5 g), JP Bupleurum Root (1.5 g), JP Mentha Herb (1.5 g), JP Angelica Dahurica Root (1.5 g), JP Saposhnikovia Root and Rhizome (1.5 g), and JP Immature Orange (1.5 g) (The Ministry of Health, Labour, and Welfare, 2021).

**TABLE 2:** Therapeutic medication for the symptoms of COVID-19 infection.

Western medication in the Control group	*N*
Dimemorfan phosphate	8
Carbocisteine	8
Acetaminophen	7
Vitamin B12	7
Tranexamic acid	5
Tulobuterol	3
Levofloxacin hydrate	2
Clarithromycin	1
Multi-ingredient cold medication	1
Thoeophyline	1
Fexofenadine Hydrochloride	1
Procaterol Hydrochloride Hydrate	1
Fluticasone Propionate Formoterol Fumarate Hydrate	1
Brotizolam	1
Difluprednate ointment	1
**Western medication in the Kampo group**	
Acetaminophen	13
Dimemorfan phosphate	7
Carbocisteine	5
Tulobuterol	2
Tranexamic acid	1
Fexofenadine Hydrochloride	1
Multi-ingredient cold medication	1
Brotizolam	1
Loxoprofen	1
Lactomin	1
**Kampo medication**	
Keigairengyoto	16
Shosaikotokakikyosekko	14
Kakkontokasenkyushin’i	11
Kakkonto	8
Gokoto	6
Saireito	3
Shosaikoto	2
Saikanto	1
Kikyosekko	1
Goreisan	1
Jinsoin	1
Chikujountanto	1
Bakumondoto	1
Tokisyakuyakusan	1

In the control group, 24 patients were treated with Western medication, and 11 patients were administered no medication. In the Kampo group, 28 patients were treated with a combination of Kampo and Western medications, and 24 patients were treated with only Kampo medication, N; number.

**FIGURE 3:**
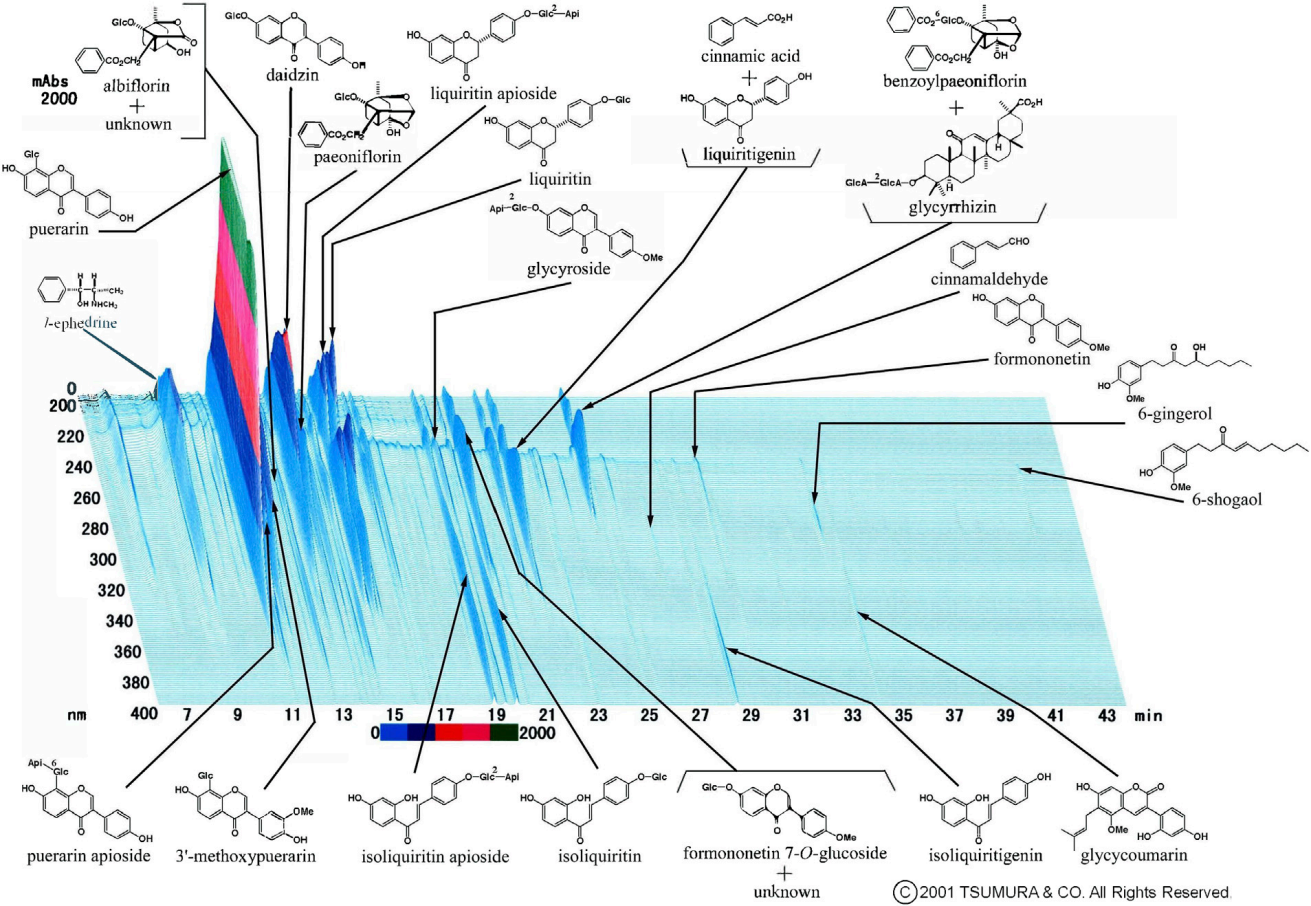
Three-dimensional high-performance liquid chromatography fingerprints of Kakkonto Extract Granules for Ethical Use (Tsumura & Co.).

**FIGURE 4:**
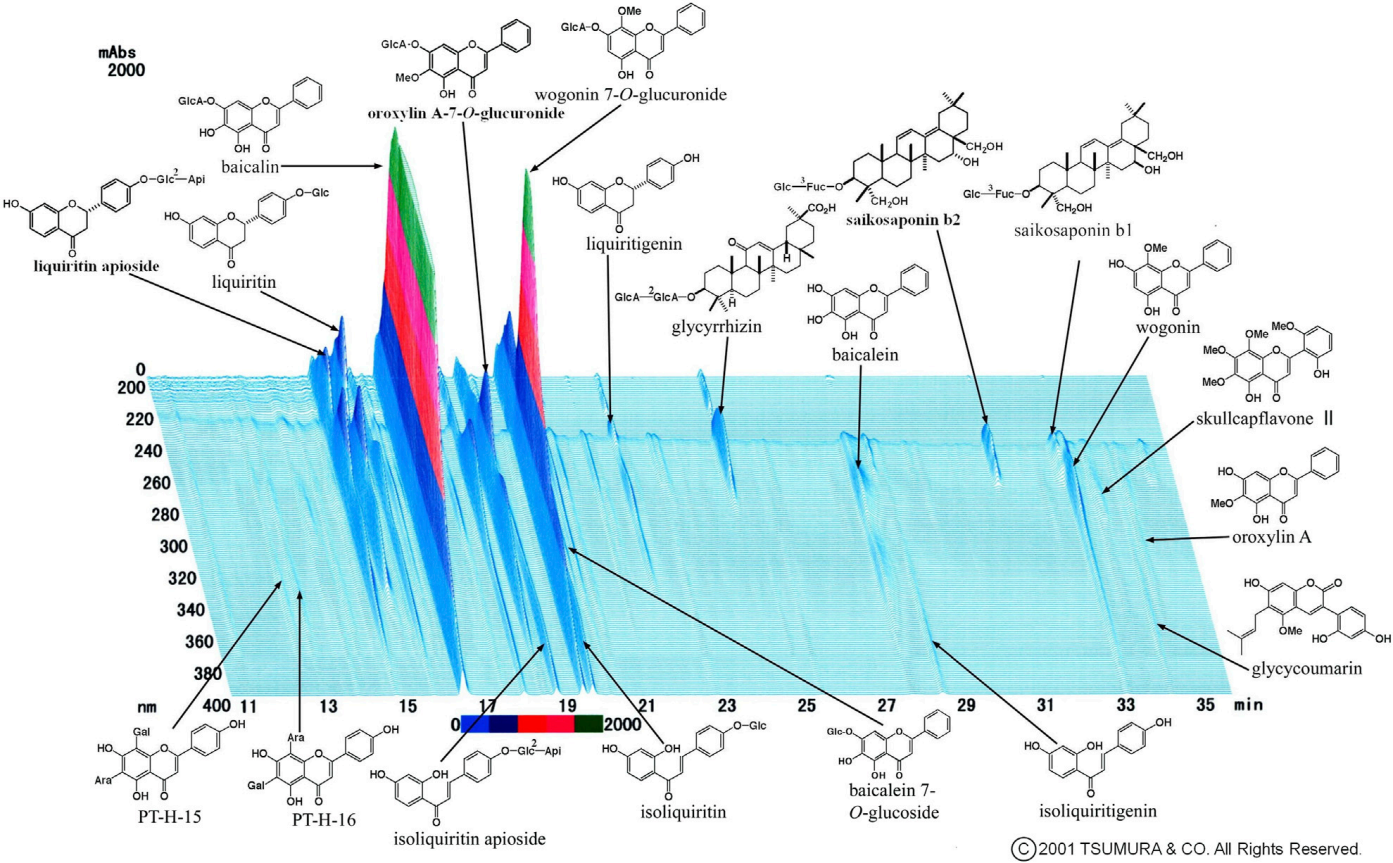
Three-dimensional high-performance liquid chromatography fingerprints of Shosaikotokakikyosekko Extract Granules for Ethical Use (Tsumura & Co.).

We occasionally used two formulas of Kampo medication in combination. This is because it was sometimes difficult to treat various conditions and progressive inflammation of COVID-19 using a single formula. The most frequently used combination was KKT and SSKKS, which was administered to eight patients (15.4%). The current prescription of KKT was KKT Extract Granules for Ethical Use (Tsumura & Co.) (Figure 3), wherein 7.5 g of KKT extract granules contain a dried extract of seven crude drugs: JP Pueraria Root (4.0 g), JP Jujube (3.0 g), JP Ephedra Herb (3.0 g), JP Glycyrrhiza (2.0 g), JP Cinnamon Bark (2.0 g), JP Peony Root (2.0 g), and JP Ginger (1.0 g). The current prescription of SSKKS was SSKKS Extract Granules for Ethical Use (Tsumura & Co.) (Figure 4), wherein 7.5 g of SSKKS extract granules contain a dried extract of nine crude drugs: JP Bupleurum Root (7.0 g), JP Scutellaria Root (3.0 g), JP Ginseng (3.0 g), JP Pinellia Tuber (5.0 g), JP Jujube (3.0 g), JP Ginger (1.0 g), JP Glycyrrhiza (2.0 g), JP Platycodon Root (3.0 g), and JP Gypsum (10.0 g).

**FIGURE 5:**
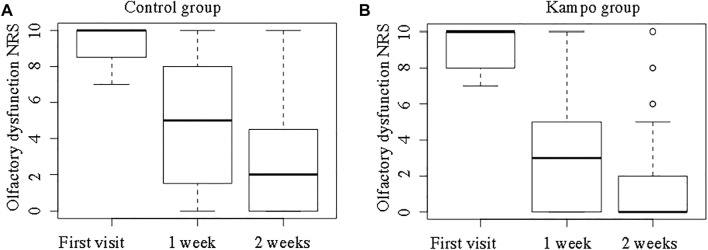
Olfactory dysfunction Numeric Rating Scale scores in the control and Kampo groups. **(A)**; Control group, **(B)**; Kampo group.

Figure 5 shows the NRS scores for OD in the two groups. The NRS scores for OD at the first visit did not differ significantly between the Kampo and control groups (median, 10 vs. 10; *p* = 0.94). The median ∆OD was greater in the Kampo group (6 vs. 8) than in the control group (3 vs. 7) both at 1 and 2 weeks after the first visit (*p* = 0.03 and *p* = 0.04, respectively).

The median ∆OD from the first visit to 1 week after the first visit was 5 (range, −2 to 10). Therefore, we defined an improvement in OD as a ∆OD ≥ 5. The univariate logistic regression analysis results demonstrated that only Kampo treatment was significantly associated with an improvement in OD [OR, 3.22 (95% CI: 1.32–8.13); *p* = 0.01]. The significant association between Kampo treatment and improvement in OD [adjusted OR, 1.27 (95% CI: 1.03–1.56); *p* = 0.03] (Table 3) was maintained in the multiple regression analysis of Kampo treatment and background factors: psychological disorder (*p* = 0.15), dysgeusia (*p* = 0.17), and days from the onset of COVID-19 to the first visit (*p* = 0.18). Age [OR, 1.02 (95% CI: 0.98–1.06); *p* = 0.32] and past history of asthma [OR, 0.59 (95% CI: 0.15–2.21); *p* = 0.41] were not significantly associated with an improvement in OD symptoms; hence, they were excluded from multiple logistic regression analysis.

**TABLE 3:** Multiple logistic regression analysis of variables affecting the improvement of olfactory dysfunction at one week after the first visit.

Item	Estimate	Standard error	T Value	Adjusted Odds ratio	95% CI	*p* Value
(Intercept)	0.767	0.185	4.141	2.15	1.50–3.10	<0.001
Psychological disorder	−0.151	0.181	−0.836	0.86	0.60–1.22	0.41
Dysgeusia	−0.018	0.128	−1.393	0.98	0.95–1.01	0.17
Days from onset of COVID-19 to medical examination	−0.022	0.018	−1.2	0.98	0.94–1.01	0.23
Kampo treatment	0.237	0.107	2.218	1.27	1.03–1.56	0.03
AIC = 118.8						

CI; confidence interval.

## Discussion

This study revealed that Kampo medicine alone or in combination with conventional treatment could promote the early recovery of OD compared with no Kampo medicine or course observation. Kampo medicine was the only factor significantly associated with reduction in OD. Currently, the recommended management of postinfectious OD is only olfactory training (Addison et al., 2021); therefore, our study may contribute to its future treatment.

COVID-19-related OD was previously considered to recover on its own with progress in time. However, recent studies revealed that nearly 50% of patients had OD more than 3 months after COVID-19 based on subjective and objective evaluation (Bussière et al., 2021; Seo et al., 2021). OD affected daily life activities (Temmel et al., 2002) and had a negative influence on psychological status (Valsamidis et al., 2020). Therefore, the prompt recovery of OD is important. Currently, no validated treatment for COVID-19-related OD exists. Only olfactory training has been suggested as an effective low-risk treatment (Whitcroft and Hummel, 2020; Seo and Lee, 2021). Oral and topical corticosteroids have been used as therapeutic approaches for OD; however, corticosteroids are not recommended for COVID-19-related OD owing to unclear evidence and potential risk of harm (Whitcroft and Hummel, 2020).

The nasal cavity is the first barrier to protect the lower respiratory tract from pathogens. SARS-CoV-2 uses human angiotensin-converting enzyme 2 (ACE2) receptors for host cell entry, mainly through endocytosis, and uses transmembrane serine protease 2 (TMPRSS2) for S protein priming and activation (Hoffmann et al., 2020). Sustentacular cells, stem cells, and perivascular cells in the olfactory epithelium express high levels of ACE2 and TMPRSS2 (Bilinska et al., 2020; Brann et al., 2020; Fodoulian et al., 2020; Kanjanaumporn et al., 2020). Therefore, the olfactory epithelium directly undergoes extensive damage (Bryche et al., 2020). A significant inflammatory response elevates proinflammatory cytokine levels, resulting in the infiltration of activated immune cells that interfere with the olfactory nervous system (Kanjanaumporn et al., 2020), which inhibits the differentiation of stem cells into olfactory receptor neurons (Chen et al., 2019). The levels of viral RNA in the olfactory bulb were higher than those in other brain regions. Moreover, elevated immunoreactivity by SARS-CoV-2 was detected as microthrombi and subsequent acute territorial brain infarcts (Meinhardt et al., 2021). Patients with COVID-19 exhibited localized abnormalities that suggest selective susceptibility of the olfactory-eloquent brain region (Kandemirli et al., 2021; Tsivgoulis et al., 2021). Prolonged OD is associated with central damage.

The mechanism of Kampo medicine-mediated early recovery from COVID-19-related OD remains unknown. However, several crude drugs of Kampo medicine reportedly possess various therapeutic effects, including anti-inflammatory and immunomodulatory effects, enhanced circulation, and nerve protection. KRT, the most frequently used Kampo medicine in this study, is used to treat inflammatory diseases of the nose, throat, and skin. Moreover, KKT in combination with SSKKS was frequently used in our study; it is used to treat common cold, fever, headache, neck stiffness, and diarrhea. SSKKS, which is composed of shosaikoto and Platycodon Root and Gypsum, is used to treat pharyngeal tonsillitis.

KRT and KKT in combination with SSKKS are used in the more progressive inflammatory phase. We reported that KKT in combination with SSKKS has symptom-relieving effects, as well as antiviral, anti-inflammatory, immunomodulatory, and antioxidant activities (Arita et al., 2020). There is little basic research on KRT. However, its component crude drugs have been reported to possess antiviral, immunomodulatory, anti-inflammatory, organ protective, and antioxidant properties.

The common crude drugs in the two prescriptions are Glycyrrhiza, Scutellaria Root, Bupleurum Root, Platycodon Root, and Paeonia Root. Glycyrrhiza inhibits viral entry, prevents internalization and replication via the action of ACE2 and 3CL hydrolase, and regulates the immune system, inflammation, cellular processes, and endocrine system in silico (Ren et al., 2020). Glycyrrhizin, a component of Glycyrrhiza, possesses glucocorticoid-like effects and inhibits nuclear factor-kappa B-dependent transcription in the nucleus (Takei et al., 2008). Glycyrrhizin reduces viral protein-induced lung cell pyroptosis and activation of macrophages and attenuates the release of proinflammatory cytokines by inhibiting high mobility group box protein one and ferritin (Gowda et al., 2021). Scutellaria Root may effectively prevent SARS-CoV-2 infection by inhibiting ACE2, 3CL, and TMPRSS2 (Ren et al., 2020; Huang et al., 2021; Liu et al., 2021). *In vivo*, baicalein, the bioactive compound of Scutellaria Root, significantly inhibited weight loss and replication of the virus in the lung tissue and mitigated lung injury attributed to SARS-CoV-2 (Song et al., 2021). Recently, the antiviral activity of baicalin (aglycone of baicalin) has been widely investigated to explore its molecular mechanisms of action, which were mainly regulation of the Janus kinase–signal transducer and activator of transcription, toll-like receptor, and nuclear factor-kappa B pathways (Li et al., 2021). Platycodin D, the bioactive compound of Platycodon Root, effectively inhibited SARS-CoV-2 entry into ACE2-expressing cells and ACE2- and TMPRSS2-expressing cells with much higher potency than existing drug candidates, such as chloroquine, camostat, and nafamostat, which may be ineffective for TMPRSS2-negative cells (Kim et al., 2021).

Paeonia Root has not been studied with respect to SARS-CoV-2. However, paeoniflorin, the bioactive compound of Paeonia Root, has been shown to inhibit lung inflammation and fibrosis in influenza A virus-induced acute lung injury, which may be associated with its regulation of the αvβ3/transforming growth factor-β1/Smads signaling pathway that regulates vascular growth and permeability, tissue inflammation, and fibrosis (Yu et al., 2021). Paeoniflorin was reported to have vasoprotective and neuroprotective properties (Wang et al., 2013; Chen et al., 2018) that could inhibit the progression of central nerve damage.

KKTSS is used for nasal congestion and rhinitis in the early inflammatory phase. This prescription also comprises Glycyrrhiza and Paeonia Root.

Based on the above, the following were considered potential mechanisms for the faster recovery in the Kampo group. Anti-SARS-CoV-2-acting crude drugs contained in Kampo medicine could reduce the viral load earlier than Western medication; moreover, the anti-inflammatory and tissue protective properties of the contained crude drugs may reduce damage to the olfactory system. When Kampo medicine is selected according to symptoms and the infectious phase, a beneficial immune response is induced. Thus, Kampo medicine as complementary treatment for COVID-19 is considered for prompt recovery of OD. Various formulas were used for each patient. Therefore, effective Kampo medicine for COVID-19-related OD cannot be determined. Additionally, Kampo medicine is considered less effective in cases of inappropriate use or significant olfactory damage.

A limitation of this study was that clinical treatment was administered at an isolation facility for patients with COVID-19 and not in a hospital. Therefore, there was a lack of detailed clinical data, the follow-up period was limited to 2 weeks, the evaluation was based on subjective symptoms only, and accurate examination was limited owing to the prevention of infection among physicians. In addition, this was a retrospective observational study, which did not include a controlled study design. The dataset was retrieved from the medical records of details on usual treatment. The treatment was administered under the medical insurance system in Japan. Further, the patient allocation to the Kampo and control groups was not randomized; therefore, patient background, including the past history of asthma, was not uniform. Thus, prospective studies are warranted to address these limitations. We are currently conducting multicenter clinical trials to demonstrate the efficacy and safety of Kampo medicine for COVID-19 (Takayama et al., 2020; Takayama et al., 2021b; Namiki et al., 2021).

## Conclusion

This study suggests that Kampo medicine for COVID-19 patients may promote the recovery of OD. The treatment of COVID-19-related OD is not yet established; therefore, preventing the prolongation of symptoms may be significant. A prospective comparative study is needed to clarify the effect of Kampo treatment, with a detailed investigation of the changes in symptoms and a long-term observation period (Takayama et al., 2021c).

## Data Availability

The original contributions presented in the study are included in the article/Supplementary Material, further inquiries can be directed to the corresponding author.

## Abbreviations

ACE2, human angiotensin-converting enzyme two; CI, confidence interval; COVID-19, coronavirus disease 2019; JP, japanese pharmacopoeia; KKT, kakkonto; KRT, keigairengyoto; KTSS, kakkontokasenkyushin’i; NRS, numeric rating scale; OD, olfactory dysfunction; ∆OD, reduction in OD score; OR, odds ratio; SARS-CoV-2, severe acute respiratory syndrome coronavirus two; SSKKS, shosaikotokakikyosekko; SST, shosaikoto; MPRSS2, transmembrane serine protease 2.
