# Supplementary material for: Kampo Medicine Promotes Early Recovery From Coronavirus Disease 2019-Related Olfactory Dysfunction: A Retrospective Observational Study
Source: Front Pharmacol. 2022 Mar 30;13:844072. doi: 10.3389/fphar.2022.844072 (PMC9006147; doi:10.3389/fphar.2022.844072)
Supplement: Supplementary file 1 [file Table1.pdf]

Supplementay Table. Kampo medication, ingredient crude drugs, and their Latin names and parts

1. Gokoto

Ingredient crude drug (English)	Amount (g)	Latin name (family name)	Part (Latin)
JP Gypsum	10.0 g	*Gypsum fibrosum*	-
JP Apricot Kernel	4.0 g	*Prunus armeniaca* Linné, *Prunus armeniaca* Linné var. *ansu* Maximowicz or *Prunus sibirica* Linné (*Rosaceae*)	*Semen*
JP Ephedra Herb	4.0 g	*Ephedra sinica* Stapf, *Ephedra intermedia* Schrenk et C. A. Meyer, or *Ephedra equisetina* Bunge (*Ephedraceae*)	*Herba*
JP Mulberry Bark	3.0 g	*Morus alba* Linné (*Moraceae*)	*Cortex*
JP Glycyrrhiza	2.0 g	*Glycyrrhiza uralensis* Fischer or *Glycyrrhiza glabra* Linné (*Leguminosae*)	*Radix*

2. Kakkonto (KKT)

Ingredient crude drug (English)	Amount (g)	Latin name (family name)	Part (Latin)
JP Pueraria Root	4.0 g	*Pueraria lobata* Ohwi (*Leguminosae*)	*Radix*
JP Jujube	3.0 g	*Zizyphus jujuba* Miller var. *inermis* Rehder (*Rhamnaceae*)	*Fructus*
JP Ephedra Herb	3.0 g	*Ephedra sinica* Stapf, *Ephedra intermedia* Schrenk et C. A. Meyer, or *Ephedra equisetina* Bunge (*Ephedraceae*)	*Herba*
JP Glycyrrhiza	2.0 g	*Glycyrrhiza uralensis* Fischer or *Glycyrrhiza glabra* Linné (*Leguminosae*)	*Radix*
JP Cinnamon Bark	2.0 g	*Cinnamomum cassia* Blume (*Lauraceae*)	*Cortex*
JP Peony Root	2.0 g	*Paeonia lactiflora* Pallas (*Paeoniaceae*)	*Radix*
JP Ginger	2.0 g	*Zingiber officinale* Roscoe (*Zingiberaceae*)	*Rhizoma*

3. Kakkontokasenkyushin'i (KKTSS)

Ingredient crude drug (English)	Amount (g)	Latin name (family name)	Part (Latin)
JP Pueraria Root	4.0 g	*Pueraria lobata* Ohwi (*Leguminosae*)	*Radix*
JP Jujube	3.0 g	*Zizyphus jujuba* Miller var. *inermis* Rehder (*Rhamnaceae*)	*Fructus*
JP Ephedra Herb	3.0 g	*Ephedra sinica* Stapf, *Ephedra intermedia* Schrenk et C. A. Meyer, or *Ephedra equisetina* Bunge (*Ephedraceae*)	*Herba*
JP Glycyrrhiza	2.0 g	*Glycyrrhiza uralensis* Fischer or *Glycyrrhiza glabra* Linné (*Leguminosae*)	*Radix*

JP Cinnamon Bark	2.0 g	*Cinnamomum cassia* Blume (*Lauraceae*)	*Cortex*
JP Peony Root	2.0 g	*Paeonia lactiflora* Pallas (*Paeoniaceae*)	*Radix*
JP Magnolia Flower	2.0 g	*Magnolia salicifolia* Maximowicz, *Magnolia kobus* De Candolle, *Magnolia biondii* Pampanini, *Magnolia*	*Flos*
JP Cnidium Rhizome	2.0 g	*Cnidium officinale* Makino (*Umbelliferae*)	*Rhizoma*
JP Ginger	1.0 g	*Zingiber officinale* Roscoe (*Zingiberaceae*)	*Rhizoma*

4. Keigairengyoto (KRT)

Ingredient crude drug (English)	Amount (g)	Latin name (family name)	Part (Latin)
JP Scutellaria Root	1.5 g	*Scutellaria baicalensis* Georgi (*Labiatae*)	*Radix*
JP Phellodendron Bark	1.5 g	*Phellodendron amurense* Ruprecht, or *Phellodendron chinense* Schneider (*Rutaceae*)	*Cortex*
JP Coptis Rhizome	1.5 g	*Coptis japonica* Makino, *Coptis chinensis* Franchet, *Coptis deltoidea* C.Y. Cheng et Hsiao, or *Coptis teeta*	*Radix*
JP Platycodon Root	1.5 g	*Platycodon grandiflorum* A. De Candolle (*Campanulaceae*)	*Radix*
JP Immature Orange	1.5 g	*Citrus aurantium* Linné var. *daidai* Makino, *Citrus aurantium* Linné, or *Citrus natsudaidai* Hayata	*uctus immaturus*
JP Schizonepeta Spike	1.5 g	*Schizonepeta tenuifolia* Briquet (*Labiatae*)	*Spica*
JP Bupleurum Root	1.5 g	*Bupleurum falcatum* Linné (*Umbelliferae*)	*Radix*
JP Gardenia Fruit	1.5 g	*Gardenia jasminoides* Ellis (*Rubiaceae*)	*Fructus*
JP Rehmannia Root	1.5 g	*Rehmannia glutinosa* Liboschitz var. *purpurea* Makino, or *Rehmannia glutinosa* Liboschitz (*Scrophulariaceae*)	*Radix*
JP Peony Root	1.5 g	*Paeonia lactiflora* Pallas (*Paeoniaceae*)	*Radix*
JP Cnidium Rhizome	1.5 g	*Cnidium officinale* Makino (*Umbelliferae*)	*Rhizoma*
JP Japanese Angelica Root	1.5 g	*Angelica acutiloba* Kitagawa, or *Angelica acutiloba* Kitagawa var. *sugiyamae* Hikino (*Umbelliferae*)	*Radix*
JP Mentha Herb	1.5 g	*Mentha arvensis* Linné var. *piperascens* Malinvaud (*Labiatae*)	*Herba*
JP Angelica Dahurica Root	1.5 g	*Angelica dahurica* Bentham et Hooker filius ex Franchet et Savatier (*Umbelliferae*)	*Radix*
JP Saposhnikovia Root and Rhizome	1.5 g	*Saposhnikovia divaricata* Schischkin (*Umbelliferae*)	*adix et Rhizoma*
JP Forsythia Fruit	1.5 g	*Forsythia suspensa* Vahl (*Oleaceae*)	*Fructus*

JP Glycyrrhiza	1.0 g	*Glycyrrhiza uralensis* Fischer, or *Glycyrrhiza glabra* Linné (*Leguminosae*)	*Radix*

5. Shosaikoto (SST)

Ingredient crude drug (English)	Amount (g)	Latin name (family name)	Part (Latin)
JP Bupleurum Root	7.0 g	*Bupleurum falcatum* Linné (*Umbelliferae*)	*Radix*
JP Pinellia Tuber	5.0 g	*Pinellia ternata* Breitenbach (*Araceae*)	*Tuber*
JP Scutellaria Root	3.0 g	*Scutellaria baicalensis* Georgi (*Labiatae*)	*Radix*
JP Jujube	3.0 g	*Zizyphus jujuba* Miller var. *inermis* Rehder (*Rhamnaceae*)	*Fructus*
JP Ginseng	3.0 g	*Panax ginseng* C. A. Meyer (*Panax schinseng* Nees) (*Araliaceae*)	*Radix*
JP Glycyrrhiza	2.0 g	*Glycyrrhiza uralensis* Fischer or *Glycyrrhiza glabra* Linné (*Leguminosae*)	*Radix*
JP Ginger	1.0 g	*Zingiber officinale* Roscoe (*Zingiberaceae*)	*Rhizoma*

6. Shosaikotokakikyosekko (SSKKS)

Ingredient crude drug (English)	Amount (g)	Latin name (family name)	Part (Latin)
JP Gypsum	10.0 g	*Gypsum fibrosum*	-
JP Bupleurum Root	7.0 g	*Bupleurum falcatum* Linné (*Umbelliferae*)	*Radix*
JP Pinellia Tuber	5.0 g	*Pinellia ternata* Breitenbach (*Araceae*)	*Tuber*
JP Scutellaria Root	3.0 g	*Scutellaria baicalensis* Georgi (*Labiatae*)	*Radix*
JP Platycodon Root	3.0 g	*Platycodon grandiflorum* A. De Candolle (*Campanulaceae*)	*Radix*
JP Jujube	3.0 g	*Zizyphus jujuba* Miller var. *inermis* Rehder (*Rhamnaceae*)	*Fructus*
JP Ginseng	3.0 g	*Panax ginseng* C. A. Meyer (*Panax schinseng* Nees) (*Araliaceae*)	*Radix*
JP Glycyrrhiza	2.0 g	*Glycyrrhiza uralensis* Fischer or *Glycyrrhiza glabra* Linné (*Leguminosae*)	*Radix*
JP Ginger	1.0 g	*Zingiber officinale* Roscoe (*Zingiberaceae*)	*Rhizoma*

Each Kampo medication used in this study was extract granules, which were manufactured from multiple crude drugs through the process of decoction, concentration, drying, and the addition of an excipient. Amount (g) means the quantity of crude drug included in the daily amount of extract granules. Detailed information on each medication is available on STORK (http://mpdb.nibiohn.go.jp/stork/)

JP: The Japanese Pharmacopoeia 17th edition English version.
